# Pretargeted radioimmunotherapy in the treatment of metastatic medullary thyroid cancer

**DOI:** 10.3747/co.v16i5.464

**Published:** 2009-09

**Authors:** F. Kraeber–Bodéré, D.M. Goldenberg, J.F. Chatal, J. Barbet

**Affiliations:** * Nuclear Medicine Department, University Hospital, Nantes, France; † Cancer Research Center, University of Nantes, INSERM, Nantes, France; ‡ Garden State Cancer Center, Center for Molecular Medicine and Immunology, Belleville, New Jersey, U.S.A; § Arronax Cyclotron, University of Nantes, Nantes, France

**Keywords:** Pretargeted radioimmunotherapy, medullary thyroid cancer, calcitonin

## INTRODUCTION

Medullary thyroid carcinoma (mtc) is a rare cancer (less than 8% of all thyroid cancers) that occurs both as a familial and as a sporadic disease. Total thyroidectomy with dissection of the ipsilateral and central lymph nodes, sometimes extended to contralateral dissection, is the primary treatment of hereditary and sporadic mtc alike. After surgery, many patients are cured, especially those with familial mtc, who are diagnosed early and without metastatic tumour spread. However, some patients show persistent disease after primary surgery, as shown by measurable serum calcitonin 1.

Three months after surgery, serum calcitonin levels are not detectable in more than 60% of patients without lymph node involvement, as compared with fewer than 20% of patients with lymph node spread 2. For relapses localized in the neck or mediastinum, a new surgical resection is usually proposed, but this treatment is followed by undetectable levels of serum calcitonin in fewer than one third of patients 3. The overall survival (os) rate 10 years after primary surgery in all patients with mtc is 69%, which decreases to 10% when distant metastases are present 4. Patients with localized or metastatic disease may survive for some years or may rapidly progress and die. Consequently, highly reliable prognostic indicators are needed for the early detection of high-risk patients who require treatment as compared with low-risk patients who warrant a “watch-and-wait” approach. Moreover, these prognostic factors can be very useful in the development of new therapeutic modalities.

Here, we consider the most appropriate prognostic indicators and the most efficient imaging techniques for the selection of patients intended to be treated with pretargeted radioimmunotherapy (prit). Then, we analyze the effectiveness and toxicity of this new therapeutic modality and compare it with alternative treatments currently available or in evaluation.

## SELECTION OF HIGH-RISK PATIENTS WHO NEED TO BE TREATED

Advanced age and advanced disease stage, with associated multiple endocrine neoplasia 2B, are commonly accepted factors of poor prognosis in mtc 5. Furthermore, the presence of node metastases has been reported to be the most significant prognostic factor 6. Kebebew *et al.* 7 stated that the European Organization for Research and Treatment of Cancer prognostic scoring system 8, which takes into account age, sex, and nature and stage of the disease, had the most effective predictive value.

Mutations in the *ret* oncogene are frequently observed in familial disease and in 43% of sporadic cases 9. Lower survival rates are associated with these mutations. The Cdc25b phosphatase has also been presented as a new indicator of aggressive mtc 10. However, although these prognostic factors and scoring systems are rather good predictors of probability of cure after primary surgery, they are not effective in predicting disease outcome for patients who are not cured after surgery.

Tumour aggressiveness has been related to the tumour cell proliferation index provided by the measure of Ki67 expression, described as another prognostic factor 11, and can also be approached by monitoring serum calcitonin or carcinoembryonic antigen (cea) concentration kinetics and by calculating doubling time (dt). We have shown that calcitonin dt is an independent predictor of survival, with a high predictive value, in patients with measurable serum calcitonin, even after repeated surgery 12. At the end of that study, 41 patients with a calcitonin dt greater than 2 years were still alive 2.9 years to 29.5 years after their initial surgery. Deaths from mtc were recorded in 20 patients, among whom 8 (67%) with a calcitonin dt between 6 months and 2 years died 40–189 months after surgery. All 12 patients with a calcitonin dt below 6 months died of their disease 6 months to 13.3 years after their initial surgery. Consequently, calcitonin dt was used to select patients with progressive disease in two clinical trials of rit.

The rit trials showed a significant increase in os as compared with a historical untreated control group matched for calcitonin dt 13. Calcitonin dt was also taken into account in a positron-emission tomography (pet) imaging study that concluded that the maximum standard uptake value (suv_max_) correlated with calcitonin dt, and that combined fluorodeoxyglucose (fdg) pet – computed tomography (ct) could be used for staging patients with progressive mtc, with possible prognostication by suv quantification 14. The suv_max_ correlated significantly with calcitonin dt (*p* = 0.011) and with minimal dt (the minimum of cea and calcitonin dt, *p* = 0.027).

Several imaging methods may be used for patients with rapidly progressing metastatic mtc before any treatment: ultrasonography and ct for neck exploration and ct for chest, abdomen, and pelvis. Moreover, we showed that magnetic resonance imaging (mri) appears to be a sensitive technique for detecting tumour spread to bone or bone marrow, with a higher sensitivity than that for bone scintigraphy 15. We also showed that the sensitivity of fdg pet–ct in progressive metastatic mtc patients was 83% for neck, 85% for mediastinum, 75% for lung, 60% for liver, and 67% for bone metastases, with an overall sensitivity of 76%.

## PRETARGETED RADIOIMMUNOTHERAPY

For radioresistant solid tumours such as mtc, pretargeted rit (prit) techniques have been developed to increase the therapeutic index over rit using directly labelled antibodies and to increase the absorbed dose delivered to tumour cells 16. An unlabelled antitumour immunoconjugate is injected first. Later, when the immunoconjugate has cleared sufficiently from the circulation, the radionuclide, coupled to a rapidly clearing agent with a high affinity for the immunoconjugate prelocalized in the tumour, is injected. Among other alternative techniques, the Affinity Enhancement System uses a bi-specific antibody and a radiolabelled bivalent hapten. In this system, the affinity of the hapten for the bi-specific antibody is limited (*K_d_* = 10^−8^ mol/L), but the bivalent hapten binds avidly to the immunoconjugate bound to the surface of target cells. The hapten–bi-specific antibody complexes in the circulation dissociate and excess hapten is cleared, at least in part, through the kidneys.

In a first clinical study, dosimetric results showed that small mtc tumours received potentially tumoricidal irradiation (up to 4.7 cGy per megabecquerel), a dose comparable to that delivered by ^131^I therapy to metastases of differentiated thyroid carcinoma (1.2–3.8 cGy per megabecquerel for tumours of 8–40 g) 17.

In 1996, a phase i/ii clinical trial then used a murine bi-specific antibody and a bivalent indium– dtpa hapten labelled with ^131^I to evaluate toxicity, pharmacokinetics, dosimetry, and antitumour activity in 26 patients with recurrence of mtc 18. The dose-limiting toxicity was hematologic, and the maximal tolerated activity was estimated at 1.8 GBq/m^2^ in the group of patients with suspected bone marrow involvement. Some therapeutic responses were observed, mainly in patients with a small tumour burden and after repeated courses of rit.

Because of the relatively high hematologic toxicity and frequent immune responses, further optimization included development of chimeric and humanized bi-specific antibodies. To determine optimal bi-specific antibody dose, hapten activity, and pretargeting interval, a prospective phase i optimization study was performed in 34 patients with cea-expressing tumours 19–20. A bi-specific antibody dose of 40 mg/m^2^, with a pretargeting interval of 5 days, appeared to be a good compromise between toxicity and efficacy. Human anti-mouse antibody elevation was observed in 8% of patients, and human anti-human antibody in 33%.

Six years after the first prit phase i/ii study and 3 years after the second, long-term disease stabilization was observed in 53% of the mtc patients, as documented by morphologic imaging (ct, mri) and serial serum calcitonin and cea measurements. A retrospective study was therefore conducted to compare survival in 29 patients given prit with survival in 39 contemporaneous untreated patients for whom data were collected by the French Tumor Endocrine Group 13. A second objective was to examine whether post-prit variation in calcitonin dt could be used as a surrogate marker for survival by comparing, among treated patients, the survival of biologic responders and non-responders. A responder was defined as an individual showing at least a 100% increase in calcitonin dt. Overall survival was significantly longer in high-risk (calcitonin dt < 2 years) treated than in high-risk untreated patients (median os: 110 months vs. 61 months; *p* < 0.030). The 47% of patients defined as biologic responders experienced significantly longer survival than did the non-responders (median os: 159 months vs. 109 months; *p* < 0.035) or untreated patients (median os: 159 months vs. 61 months; *p* < 0.010). Treated patients with bone or bone marrow disease had a longer survival than did patients without such involvement (10-year os: 83% vs. 14%; *p* < 0.023). Toxicity was mainly hematologic and related to bone or bone marrow tumour involvement, which was shown by our group to be more frequent in patients with metastatic mtc than had been previously reported 15. Indeed, in the two phase i/ii studies, mri confirmed bone or bone marrow involvement in most patients with bone or bone marrow activity uptake observed by scintigraphy 13. Consequently, the observed hematologic toxicity was expected in such patients who had the best response rate, probably because their bone marrow involvement illustrated the situation of disseminated microscopic disease commonly considered as the most favourable indication for rit.

Only 3 cases of myelodysplasia were observed in our series of 70 mtc patients treated with prit. These 3 patients were heavily pretreated, in particular with external radiotherapy. No renal toxicity was reported after prit.

Following the encouraging results obtained in the two phase i/ii studies, a phase ii prit study was undertaken to evaluate response rate, time to progression, and os in progressive mtc patients (calcitonin dt < 5 years). Between April 2004 and January 2008, 48 patients were enrolled. So far, 45 patients have been treated, receiving 40 mg/m^2^ of a bi-specific antibody (prepared by coupling the Fab fragments of humanized anti-cea antibody hMN-14 with the Fab fragment of the mouse anti-dtpa–indium antibody 734), followed by 1.8 GBq/m^2^ of bivalent indium–dtpa hapten labelled with ^131^I given 4–6 days later. A second course of treatment has been given to 6 of the patients.

A preliminary analysis of the results was performed in September 2008 for 33 evaluable patients (20 men, 13 women) who received 35 treatments and had a median of 15 months of follow-up (range: 6–36 months). A patient was considered unresponsive if progression according to the Response Evaluation Criteria in Solid Tumors (recist) criteria, fdg pet, or serum concentration of biomarkers was observed at 3 months post-rit, or if no effect on cea or calcitonin dt (less than 100% increase of cea or calcitonin dt) was observed.

**FIGURE 1:**
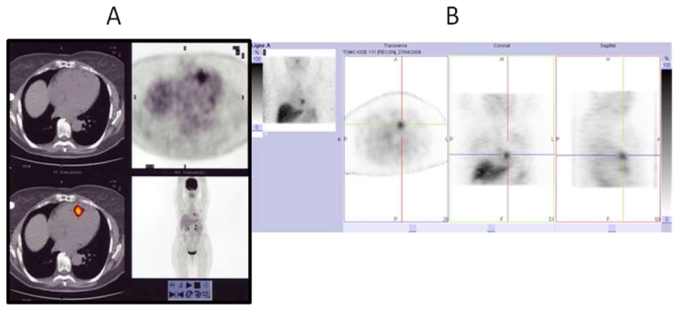
Imaging by (A) fluorodeoxyglucose positron-emission tomography and (B) immunoscintigraphy in a patient having medullary thyroid carcinoma with cardiac metastasis. The images show good tumour targeting with both radiopharmaceuticals.

In the 33 evaluable patients, the median pre-prit calcitonin dt was 1.2 years (range: 0.2–5.9 years), and cea dt was 1.8 years (range: 0.5–23.8 years). Tumour targeting was shown in all cases. The sensitivity of scintigraphic imaging was 92%. Figure 1 shows the high tumour uptake observed in a mtc patient with a cardiac metastasis. Allergic reactions were observed during 2 bi-specific antibody infusions, grade 1 liver toxicity after 3 of 35 injections (8.5%), and grade 3 or 4 hematologic toxicity after 19 of 35 injections (54%). Efficacy was observed after 18 of 35 prit injections (51%), with a time to progression of 18 months (range: 6–36 months) by recist criteria and 15 months (range: 6–36 months) by pet and biomarker levels.

In this series, 24% of patients (8/33) were considered to be low risk (dt > 2 years), 39% (13/33) to be intermediate risk (dt: 6 months–2 years), and 18% (6/33) to be high risk (dt < 6 months). Efficacy was found in 62% of patients in the low-risk group, 53% in the intermediate-risk group, and 50% in the high-risk group.

## ALTERNATIVE TREATMENT MODALITIES

The radiopharmaceutical (^90^Y–dota)–toc is another agent that has been successfully used in the treatment of endocrine gastroenteropancreatic tumours, and it has been extended to patients with mtc. In a phase ii clinical trial, 31 patients with progressive metastatic mtc were injected with a median cumulative activity of 12.6 GBq, and response was defined as a decrease in serum calcitonin after therapy 21. Interestingly, a post-therapeutic prolongation of calcitonin dt of at least 100% 12 was found in 58% of the patients, and a significantly longer survival was observed in responders than in non-responders. Unfortunately, drawing any valid conclusion with regard to a potential survival benefit is difficult because of a lack of pre-therapeutic selection of patients based on a validated prognostic factor such as calcitonin dt 12. Indeed, patients with progressive disease but a long calcitonin dt (>2–5 years), can experience very long periods of survival in the absence of treatment. Consequently, it is crucial to select patients with poor prognostic indicators before considering a potential survival benefit. Moreover, it is important to highlight that only 60%–70% of patients with mtc express somatostatin receptors, but more than 90% of patients with mtc express cea.

Clinical chemotherapy studies—limited in number, enrolling small numbers of patients, and using various drugs or combinations of drugs—were performed more than 10 years ago. In a total of 87 patients enrolled in four trials and treated with various chemotherapeutic regimens, progression-free survival (reported for only 22 patients) ranged from 4 months to 29 months (median: 10 months). Overall survival (reported for 20 patients) ranged from 8.5 months to 33 months or more (median: 17.5 months) 13. In the absence of data on pretreatment prognostic indicators of survival for treated patients, it is difficult to draw any valid conclusion on the true treatment effectiveness. Moreover, severe toxicity has been reported with some combination chemotherapy regimens 22. Consequently, chemotherapy cannot currently be considered a promising therapeutic option for patients with advanced disease.

In patients with metastases predominantly localized in the liver, selective intra-arterial chemoembolization using various drugs has been performed in 23 patients in two studies 23,24. Some transient partial remission or stabilization (median duration: 24 months), with good symptom palliation, was observed in 70% of cases (16/23). This effectiveness was observed mainly in patients with limited liver involvement (<30%). Thus, chemoembolization can be useful for a small percentage of patients with metastatic extension limited to a small part of the liver. However, in our experience, no patient had metastatic extension limited to liver 14,15.

Among signal transduction pathways that lead to neoplastic transformation, the Ret protein plays a major role in mtc 25. Consequently, Ret appears to be a favourable target for molecular therapy, even if a substantial number of patients with the sporadic form of the disease cannot benefit. Other signalling components, including receptors for vascular endothelial growth factor (vegfr), epidermal growth factor (egfr), and platelet-derived growth factor (pdgfr), can be involved in mtc, and multikinase inhibitors targeting one or some of them have been evaluated in clinical trials.

Imatinib mesylate (Gleevec: Novartis Pharmaceuticals, St. Louis, MO, U.S.A.), which inhibits Ret (among other protein tyrosine kinases) has been used in three clinical studies involving 30 patients 26–28. Stabilization was observed in 30% of patients (9/30) over 6–24 months. Severe toxicity—including rash, fatigue, laryngeal mucosal swelling, nausea, and vomiting—was reported in one study 27.

More recently, other protein kinase inhibitors have been evaluated in patients with advanced mtc, and some preliminary results are available in abstract form only. Axitinib, an inhibitor of vegfrs 1, 2, and 3, was evaluated in 60 patients with advanced thyroid cancers, including 12 patients with mtc 29. Stabilization was observed in 50% of all patients for up to 13 months, without any differentiation for patients with mtc. Sorafenib, which selectively inhibits Ret tyrosine kinase, was evaluated in 5 patients with metastatic mtc 30. Surprisingly, 1 complete response was observed, together with 1 partial response and a 50% decline of calcitonin in all 5 patients; however, the initial dose had to be reduced by 50% because of serious side effects. Vandetanib, which targets Ret, vegfr, and egfr tyrosine kinases, was evaluated in 30 patients with locally advanced or metastatic hereditary mtc 31. A partial response was observed in 20% of patients (6/30) and stabilization in 30% of patients (9/30) for up to 9 months. A biologic response was observed in 63% (19/30) for at least 6 weeks. Some grade 3 adverse events, including rash and diarrhea, were observed in 3 patients. Finally, motesanib, which targets all known vegf, pdgf, Kit, and Ret receptors, was evaluated in 83 patients with both hereditary and sporadic mtc 32. A partial response was observed in 2 patients and stabilization for no longer than 6 months in 43 (52%).

## PATIENT STRATIFICATION FOR FUTURE STUDIES

All these studies performed in patients with advanced locally recurrent or metastatic mtc and using various multikinase inhibitors have shown some tumour effect documented by a substantial decrease in calcitonin. A transient stabilization of disease was observed, extending up to more than 24 months. However, in the absence of data on real tumour growth rate before treatment, it is not possible to draw any valid conclusions about potential survival benefit.

As mentioned earlier, we showed that calcitonin dt is the best prognostic indicator for selecting patients with rapidly progressing metastatic disease before any investigational treatment 12. In patients with a calcitonin dt longer than 5 years, life expectancy is very long and watchful waiting may be the most appropriate strategy. For patients with a calcitonin dt shorter than 2 years and, all the more, shorter than 6 months, the disease is rapidly progressing, with a short life expectancy that warrants the use of investigational drugs.

## CONCLUSIONS

Currently, no drug is approved for the systemic treatment of metastatic mtc. No real survival benefit has been convincingly documented with chemotherapy, which furthermore is associated with severe toxicity. It is too early to evaluate the potential effectiveness of multikinase inhibitors. Reported results of phase ii trials have shown some transient disease stabilization, but more patients with documented rapidly progressive metastatic disease should be included and their survival compared with that of comparable untreated patients from historical studies. We appreciate that an optimal comparison would use randomized prospective trials, but because of the low frequency of mtc, too many years would be required to enrol a sufficient number of patients, especially if only patients with a short calcitonin dt are included. Additional years would be necessary for survival analyses. To this point, prit has been the only innovative treatment modality to convincingly show some survival benefit in patients with rapidly progressing metastatic disease.
